# The Correlation between the Gut Microbiota of *Endoclita signifer* (Lepidoptera, Hepialidae) Larvae and Their Host Preferences

**DOI:** 10.3390/insects14120919

**Published:** 2023-12-01

**Authors:** Jintao Lu, Xiaoyan Su, Zhende Yang, Ping Hu

**Affiliations:** 1 Guangxi Colleges and Universities Key Laboratory for Cultivation and Utilization of Subtropical Forest Plantation, Guangxi Key Laboratory of Forest Ecology and Conservation, College of Forestry, Guangxi University, Nanning 530004, China; ljt6212@st.gxu.edu.cn (J.L.);; 2 Liu Wan Forest Farm of Guangxi, Yulin 537899, China

**Keywords:** *Endoclita signifer*, gut microbiota, high-throughput sequencing, transfer host

## Abstract

**Simple Summary:**

*Endoclita signifer* (Lepidoptera, Hepialidae) poses a significant threat as a forestry pest to a diverse range of host plants. The gut microbiota of insects plays a pivotal role in establishing a symbiotic relationship with the host insect. Our research aims to scrutinize alterations in the gut microbiota and their correlation with distinct plant-feeding preferences, laying the groundwork for developing biological control strategies against *E. signifer*. We relocated *E. signifer* that had endured a period in *Eucalyptus grandis × Eucalyptus urophylla* to two different plant species, namely *Mallotus apelta* and *Broussonetia papyrifera*. Following a 4-week period, high-throughput sequencing was performed on the gut microbiota of moth larvae feeding on three plant species (*E. grandis × E. urophylla*, *M. apelta*, and *B. papyrifera*). The microbial composition in the *E. signifer* intestines exhibited similarities at the phylum level but disparities at the genus level. Concurrently, the *E. signifer* feeding preferences were predominantly linked to the nutritional composition of the plants. These findings imply that feeding preferences can impact the gut microbiota of *E. signifer*, with the nutritional composition of the plants emerging as a pivotal factor steering these preferences. This discovery introduces a novel perspective for the advancement of biological control techniques against *E. signifer*.

**Abstract:**

Insects’ gut microbiota plays a crucial role in their host selection, adaptation, and plasticity. This study explored the impact of gut bacteria on the adaptation of host selection under different stresses (diverse feeding preferences and no feeding preferences). The seventh instar *E. signifer* larvae were artificially transferred from the most-selected host *E. grandis* × *E. urophylla* (Es) to more preferred hosts, *M. apelta* (Ma), as well as the non-preferred host, *B. papyrifera* (Bp). We then obtained the larval gut of three different feeding preference hosts. The gut bacterial DNA was sequenced and analyzed based on 16S rRNA. There were significant differences in the composition of dominant gut bacteria between Es with Ma and Bp, but without significant differences between Ma and Bp. In the process, *Burkholderia* and *Microbacillus* with degrading pesticides had significant changes, and *Enterococcus* with insect gut probiotics also had significant changes. The presence of enterococcus may be one of the main causes of intestinal microbiota changes before and after host transfer. Notably, when the feeding of *E. signifer* changes, the complex connections that exist between gut bacteria also change. Additionally, there was a negative correlation between the feeding preferences of *E. signifer* and the metabolic functions of their gut bacteria. This study provided a theoretical basis for the prediction and use of gut bacteria to interfere with the feeding of *E. signifer*.

## 1. Introduction

The gut microbiota of insects and their host insects have a symbiotic relationship. The gut microbiota relies on the host to provide nutrition and a survival environment [1]. The host insect not only needs the gut microbiota to assist in the digestion and decomposition of food, but also to transform exogenous toxins into non-toxic substances through metabolism and mineralization and play a detoxifying role for the host insect. The symbiotic microbial community residing in the gut tract not only provides the host with essential nutrients for growth and development, but also assists the host in resisting invasion by parasites [2,3,4]. Research has found that the intestines of most Lepidoptera insects were mainly composed of Proteobacteria, Firmicutes, and Actinobacteria [5]. Among them, Proteobacteria and Firmicutes have a higher proportion as dominant phyla, such as *Limantria dispar*, *Helicoverpa armigera*, and *Bombyx mori* [4,6,7]. Although they were all Lepidoptera insects, the main bacterial genera in the intestines of different Lepidoptera insects may vary. For example, Enterococcus was the main bacteria in the intestines of *Spodoptera frugiperda* and *L. dispar* [6,8]. *Galleria mellonella* reveals through 16S-rDNA sequencing that the dominant bacterial genera in its larval intestines include *Enterococcus* and *Microbacterium* [9], but *Ralstonia* was one of the dominant bacterial genera at the genus level in the intestines of *B. mori* [10]. Even for the same insect, after feeding on different foods, there will be significant differences in its gut microbiota. For example, the abundance of *Burkholderia* was higher in the intestines of *Hyphantria cunea* larvae that feed on *Platanus × acerifolia* and *Diospyros kaki*; however, when the food plants changed to *Prunus armeniaca* and *Populus*, the abundance of *Enterococcus* and *Escherichia* in the intestines of *H. cunea* larvae was larger [11]. A large number of studies have shown that food is an important source of insect gut microbes and a determinant of gut microbiota structure, which has been verified in *H. armigera*, *Nasutitermes takasagoensis*, and *Cnaphalocrocis medinalis* [12,13,14]. In addition, feeding *Blattella germanica* with 0%, 24%, and 50% protein content lead to the finding that its gut microbial community was highly variable [15]. In a short period, the composition of the microbiota may be related to a specific diet.

Insects, after host switching, are in a dynamic process of adapting to new hosts, and their gut microbial populations are also in a state of mutual transformation [16]. Studies have shown that artificial feed and different combinations of commercial feed patterns have different effects on the gut microbiota of *B. mori* [10]. Under different feeding patterns (wild natural food and artificial diet), there are significant differences in the diversity and composition of the gut microbiota of *B. mori*. The longer feed time with an artificial diet, the lower its abundance. In addition, research has shown that the gut microbiota and insect hosts have a long-term co-evolutionary relationship [17]. When *Plutella xylostella* feeds on an artificial diet and is then transferred to different host plants, its larval gut microbes need a long-term adaptation process to the host plants [18]. By switching the host feeding of *Batocera lineolata*, it is difficult to change the diversity of its gut bacteria in a short time [19]. All of these studies indicate that insect gut microbes play an important role in their hosts’ adaptation.

*E. signifer* is a wood-boring pest [20]. The first and second instar larvae feed on humus in the soil, and then the third instar larvae start to climb trees and feed on plant trunks. They spin silk to weave their excrement and wood chips into a fecal bag covering the entrance of the insect tunnel, which protects the larvae and makes them very difficult to control [21]. The latest research showed that *E. signifer* has harmed 54 species of woody plants from 30 families and 42 genera in Guangxi [22]. Among them, *Eucalyptus* was its most preferred host, and *E. grandis × E. urophylla* was the most severely affected [20]. Except *Eucalyptus*, *M. apelta* was the most severely affected wild host, showing the strong preference of *E. signifer* [20]. Moraceae was still within the feeding range of *E. signifer*, but *B. papyrifera*, like *Eucalyptus*, is a fast-growing tree, favored mostly by wood-boring beetles (*Apriona germari,* etc.), and so it is not a wild host of *E. signifer* [23,24], so it can be studied as a non-host with no preference [22].

It was generally believed that gut microbes were inseparable from the normal life activities of host insects. By using insect gut microbiota as a carrier, we can study the adaptability of insects to the environment and their impact on host insects [25]. At present, there are many reports on the research of insect gut microbiota, but there are few systematic studies on the adaptation of gut microbes to their hosts after insects move to different stressful hosts. This study uses high-throughput sequencing technology to examine the diversity and species composition variations in the gut microbiota of *E. signifer* larvae feeding on three distinct host preferences. It investigates the factors influencing the adaptive preference of *E. signifer* for different hosts from a microbial standpoint. This research provides a reference for utilizing gut bacteria to interfere with this pest’s feeding behavior and establishes a foundation for future research on the host adaptation and risk assessment of *E. signifer*.

## 2. Materials and Methods

### 2.1. Test Plants and Insects

**Figure 1:**
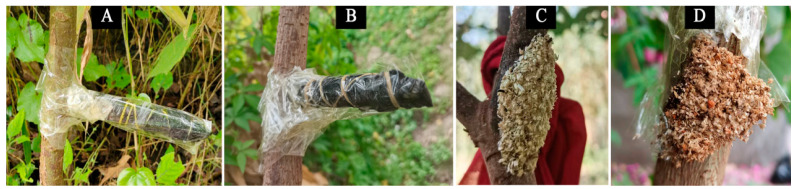
Artificial transfer: the larvae were fixed on the host plants, *M. apelta* (**A**) and *B. papyrifera* (**B**), through centrifugal tubes; the larvae formed fecal bags and showed they had transferred to the host plants *M. apelta* (**C**) and *B. papyrifera* (**D**) successfully.

The test plants were *M. apelta*, *E. grandis × E. urophylla* (located at Gao Feng Forest Farm Liuli Branch, N22°96′, E108°33′), and *B. papyrifera* (located at Guangxi University Teaching Practice Base, N22°48′, E108°22′). The 7th instar larvae of *E. signifer*, which have survived in *E. grandis × E. urophylla* for 4 instars and were in a state of complete adaptation to *E. grandis × E. urophylla*, were selected as the test insects. These test insects were all collected from the Liuli Branch. The method of transferring *E. signifer* to different hosts was according to previous studies [20,22]. The 7th instar larvae of the *E. signifer* were put into 15mL centrifuge tubes wrapped with black film in advance, and were then fixed onto the trunks of *M. apelta* and *B. papyrifera* (Figure 1A,B, respectively). After 6 weeks, when the larvae fed and survived in the trunk and formed fecal bags (Figure 1C, D), this showed that they had transferred successfully. When the transfer was successful, three larvae of the *E. signifer* that damaged the *E. grandis × E. urophylla* (Liuli Branch, Es) and were artificially transferred to *M. apelta* (Ma) and *B. papyrifera* (Bp) were taken for subsequent experiments.

### 2.2. Stripping the Gut of E. signifer Larvae

The *E. signifer* larvae (a total of 9) were placed in a −20° freezer for 10–20 min to render them unconscious. The larvae were disinfected with 75% alcohol on an ultra-clean workbench. Sterilized tweezers and scissors were used to dissect the insect body, remove the gut, and place it in 2mL centrifuge tubes. The tubes were then labeled and stored at 80 °C for future use.

### 2.3. DNA Extraction and PCR Amplification

The DNA of the larval gut microbial community was extracted according to the instructions of the FastDNA^®^ Spin Kit for Soil (MP Biomedical, Union City, CA, USA). The quality of the extracted DNA was checked via 1% agarose gel electrophoresis, and the DNA concentration and purity were measured using the NanoDrop2000. The V3-V4 variable region of the 16S rRNA gene of the sample was amplified via PCR. The upstream and downstream primers were 338F (5′-ACTCCTACGGGAGGCAGCAG-3′) and 806R (5′-GGACTACHVGGGTWTCTAAT-3′), respectively [26].

### 2.4. PCR Product Identification, Purification, and Quantification

We utilized 5 μL of the PCR amplification product for a 2% agarose gel electrophoresis procedure. We analyzed the outcomes, employing the Amersham Imager 600. Subsequently, we employed the AxyPrep DNA Gel Extraction Kit (Axygen Biosciences, Union City, CA, USA) to purify and retrieve the PCR product containing discernible bands. We then quantified the recuperated product using the Quantus™ Fluorometer (Promega, Madison, WI, USA). Upon verification of the concentration and quality, we amalgamated the retrieved products, ensuring equal concentrations and volumes.

### 2.5. Data Analysis and Statistics

The Fastp (Ver0.20.0) software was used for the quality control of the original sequencing sequences, and the FLASH (ver1.2.3) software was used for splicing [27]. The UPARSE (version 7.1) software was used for the OTU clustering of the sequences and the removal of chimeras [28]. The species classification annotation of each sequence was completed using the RDP classifier, and the Silva 16S rRNA database (v138) was used for species annotation [29]. MOTHUR and UniFrac were used to estimate the α and β diversity based on the OTUs level [30,31]. The statistical software R (version 4.1.3) was used to draw dilution curve graphs and Beta diversity analysis graphs [32]. IBM SPSS Statistics 25 was used to perform a one-way analysis of variance (one-way ANOVA) on the OTUs, Alpha indices, and abundance of functional bacterial groups in the community, where *p* < 0.05 was significant [33].To understand the relationship between genera, the Spearman correlation coefficient was used for network analysis. Gephi was used to calculate the network topological properties [34]. Prokaryotic taxon functional annotation (FAPROTAX) was used to predict the functions of microbial communities in the different samples [35]. Finally, all images were organized and typeset using Adobe Photoshop CC 2018.

## 3. Results

### 3.1. Sequencing Information and Quality Analysis of Gut Microbiota

**Figure 2:**
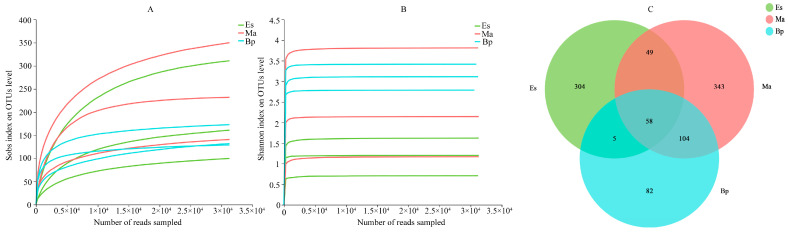
(**A**) Sobs index dilution curve of OTU levels. (**B**) Shannon index dilution curve of OUT levels. (**C**) Venn diagram of gut microbiota of larvae feeding on three hosts, and the numbers in (**C**) represent the number of OTUs. Note: Es: *E. grandis × E. urophylla*, Ma: *M. apelta*, and Bp: *B. papyrifera*.

The high-throughput sequencing results of the gut microbiota of *E. signifer* larvae feeding on three hosts (Es: *E. grandis × E. urophylla*, Ma: *M. apelta*, and Bp: *B. papyrifera*) generated a total of 347,770 original sequences. We removed sequences with low sequencing quality and those that failed in the assembly, ultimately retaining 335,074 valid sequences. The efficiency of each sample’s spliced valid sequences was greater than 90%, and the average length of the sequences exceeded 400 bp (Table 1). Both the Sobs and Shannon indexes gradually flattened with the increase in sampling read times (Figure 2A,B), indicating that the selected sequencing data volume of this experiment was reasonable and can be used for further analysis.

### 3.2. OTUs

The OTU results obtained from the clustering analysis were normalized, and a Venn diagram was drawn to intuitively display the similarity of the OTU composition between different groups. As shown in Figure 2C, the three groups of samples had a total of 58 OTUs, accounting for 4.76% of the total OTUs. The number of OTUs shared by two groups, Es and Ma, have 49 shared OTUs (accounting for 5.05% of these two groups’ OTUs); Es and Bp have five shared OTUs (accounting for 0.75% of these two groups’ OTUs), and Ma and Bp have 104 shared OTUs (accounting for 33.77% of these two groups’ OTUs). The number of unique OTUs of Es, Ma, and Bp was 304, 343, and 82, respectively, accounting for 73.08%, 61.91%, and 32.93% of their respective groups (Table 1).

### 3.3. Alpha and Beta Diversity Analysis

**Figure 3:**
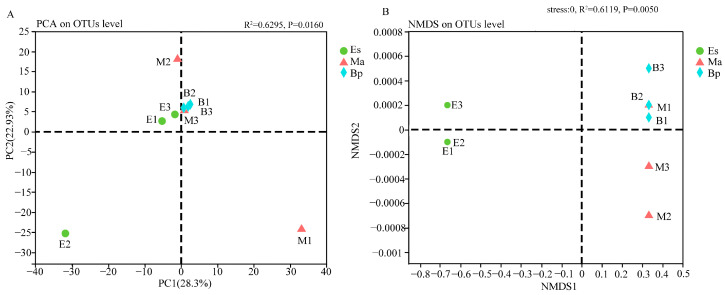
Analysis of Beta diversity of larvae enterobacteria feeding on three hosts. PCA (**A**) and NMDS (**B**) analysis of bacterial community at OTU level. Note: Es: *E. grandis × E. urophylla*, Ma: *M. apelta*, and Bp: *B. papyrifera*.

The diversity of microbes can be reflected based on the Alpha diversity index of a single sample, which indicates the richness and diversity of the microbial community. This study selected four commonly used indices (the Shannon index, the Simpson index, the Ace index, and the Chao1 index) to analyze the Alpha diversity of the gut microbiota of *E. signifer* larvae feeding on three hosts (Es, Ma, and Bp). The Alpha diversity indices of the gut microbiota of the larvae feeding on the three hosts were analyzed using a one-way analysis of variance. As shown in Table 2, there was a significant difference in the Shannon index between the samples of Es and Bp (*p* < 0.05), while there was no significant difference in the Simpson, Ace, and Chao1 index values among the three groups. Based on the OTU level, a principal component analysis (PCA) and non-metric multidimensional scaling (NMDS) based on the Bray–Curtis similarity coefficient were performed on grouped samples. The results showed that the samples of Ma and Bp intersect (Figure 3A M3~B2, Figure 3B M1~B2), but they did not coincide with the samples of Es.

### 3.4. Composition of Gut Microbiota

**Figure 4:**
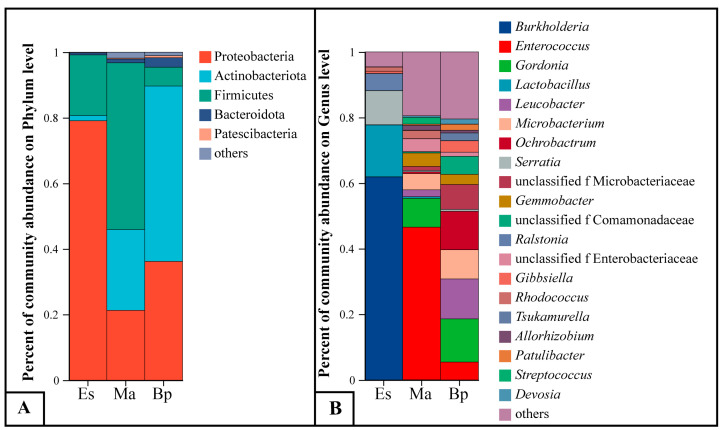
The species composition of the larval bacterial community that feed on three hosts at the phylum and genus levels. (**A**) Phylum levels. (**B**) Genus levels. Note: Es: *E. grandis × E. urophylla*, Ma: *M. apelta*, and Bp: *B. papyrifera*.

At the phylum classification level, a total of 31 phyla were annotated, including Proteobacteria, Firmicutes, Actinobacteriota, and Bacteroidota. Figure 4A shows that the dominant phyla in the samples of Es are Proteobacteria (79.06%) and Firmicutes (18.52%); the dominant phyla in the samples of Ma are Proteobacteria (21.25%), Firmicutes (50.85%), and Actinobacteriota (24.65%); and the dominant phyla in the samples of Bp were Actinobacteriota (53.48%) and Proteobacteria (36.17%).

At the genus level, a total of 462 genera were annotated (Figure 4), including *Burkholderia*, *Lactobacillus*, *Serratia*, *Enterococcus*, *Gordonia*, *Leucobacter*, *Ochrobactrum*, *Microbacterium*, *Gemmobacter*, Unclassified Microbacteriaceae, and *Ralstonia*. As seen from Figure 4B, the dominant genera in the samples of Es included *Burkholderia* (61.86%), *Lactobacillus* (15.78%), *Serratia* (10.41%), and *Ralstonia* (5.25%); the samples of Ma included *Enterococcus* (46.53%), *Gordonia* (8.78%), *Microbacterium* (4.98%), *Gemmobacter* (4.13%), *Leucobacter* (2.09%), and Unclassified Microbacteriaceae (1.20%); the dominant genera in the samples of Bp included *Gordonia* (13.18%), *Leucobacter* (12.19%), *Ochrobactrum* (11.73%), *Microbacterium* (8.89%), Unclassified Microbacteriaceae (7.61%), *Enterococcus* (5.39%), and *Gemmobacter* (3.10%).

### 3.5. Inter-group Species Diversity Analysis

**Figure 5:**
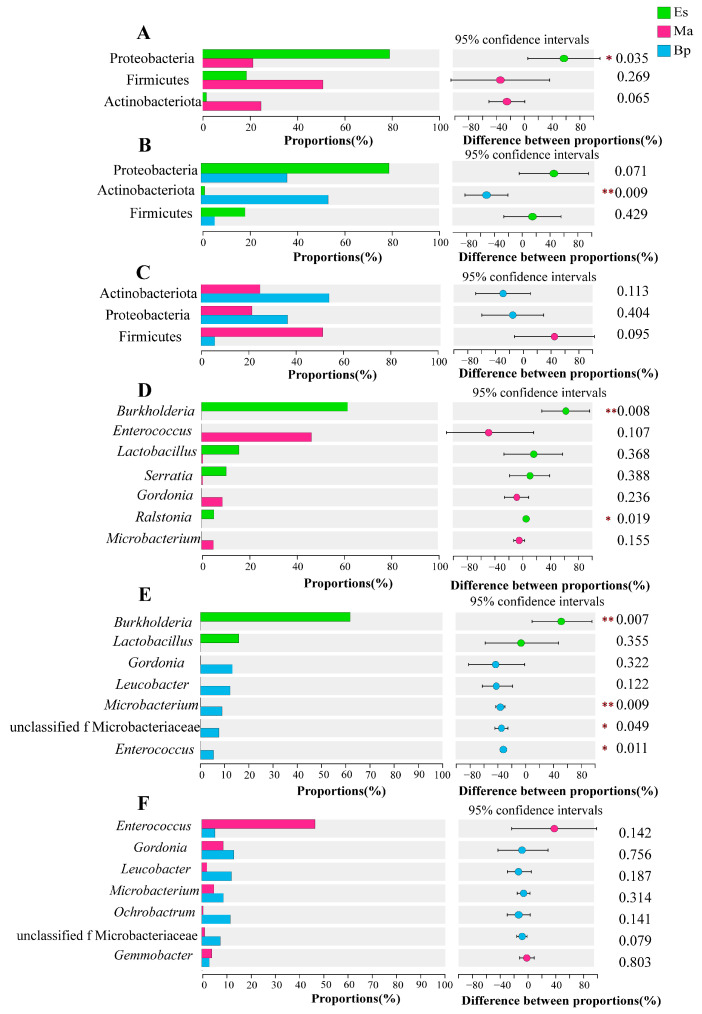
Analysis of the relative abundance differences in the larval bacterial community structure that feed on three hosts at the phylum and genus levels. (**A**) Feeding on Es and Ma at phylum levels. (**B**) Feeding on Es and Bp at phylum levels. (**C**) Feeding on Ma and Bp at phylum levels. (**D**) Feeding on Es and Ma at genus levels. (**E**) Feeding on Es and Bp at genus levels. (**F**) Feeding on Ma and Bp at the genus levels. Note: “*” indicates significant difference (*p* < 0.05); “**” indicates extremely significant difference (*p* < 0.01). Es: *E. grandis × E. urophylla*, Ma: *M. apelta*, and Bp: *B. papyrifera*.

At the phylum level, the dominant bacterial phyla of each group were analyzed forinter-group differences using Student’s *t* test. The abundance of Proteobacteria in the Es was significantly higher than that in the Ma (*p* = 0.035 < 0.05, Figure 5A); the abundance of Actinobacteria in the Bp samples was significantly higher than that in the Es (*p* = 0.009 < 0.01, Figure 5B); there was no significant difference in the abundance of dominant bacterial phyla at the phylum level between the Ma and Bp (Figure 5C).

At the genus level, the dominant genera of each group were analyzed for inter-group differences using Student’s *t* test. As shown in Figure 5D, the abundance of *Burkholderia* in the Es was significantly higher than that in the Ma (*p* = 0.008 < 0.01), and the abundance of *Ralstonia* in the Es was significantly higher than that in the Ma (*p* = 0.019 < 0.05). As shown in Figure 5E, the abundance of *Burkholderia* in the Es was significantly higher than that in the Bp (*p* = 0.008 < 0.01), and the abundance of *Microbacterium* in the Ma was significantly higher than that in the Es (*p* = 0.0095 < 0.01), and both unclassified Microbacteriaceae and *Enterococcus* in the Bp were significantly more abundant than those in Es (*p* = 0.050, 0.011 < 0.05). As shown in Figure 5F, at the genus level, there was no significant difference between Ma and Bp samples in terms of the dominant genera.

### 3.6. KEGG Function Prediction of Gut Microbiota

**Figure 6:**
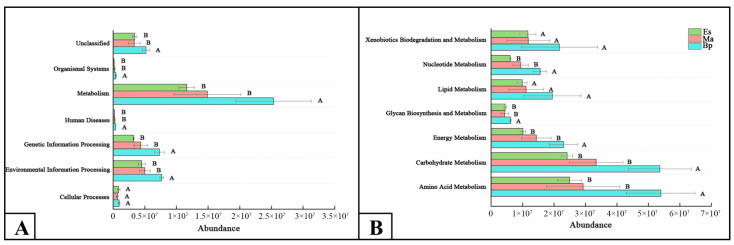
(**A,B**) Abundance of gut microbiota of larvae feeding on three hosts at the KEGG level. A represents the abundance at the KEGG primary pathway level, and B represents the abundance at the KEGG secondary pathway level. Species relationships among gut microbiota. Note: Es: *E. grandis × E. urophylla*, Ma: *M. apelta*, and Bp: *B. papyrifera*.

The abundance of each microbial community in the gut of *E. signifer* larvae feeding on three hosts (Es, Ma, and Bp) was predicted at different KEGG (Kyoto Encyclopedia of Genes and Genomes, www.genome.net (accessed on 18 October 2023)) pathway levels using PICRUSt. As depicted in Figure 6A, among the primary pathways, the abundance of functional microbial communities was significantly higher in Bp than in Es and Ma (*p* < 0.05), except for cellular processes. These communities were mainly enriched in metabolism. Further analysis of the secondary metabolic pathways of these three groups are shown in Figure 6B, and revealed that the abundance of functional microbial communities was primarily concentrated in carbohydrate metabolism and amino acid metabolism at the secondary pathway level. In these two metabolic pathways, the abundance of functional microbial communities in Bp was also significantly higher than that in Es and Ma (*p* < 0.05).

### 3.7. Species Relationships among Intestinal Flora

**Figure 7:**
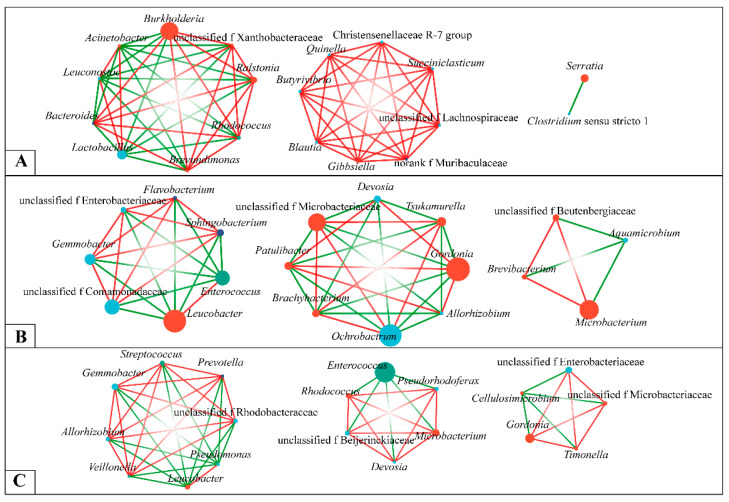
Network diagram of dominant genera (top 20) of intestinal microbial of larvae feeding on three hosts. (**A**) Feeding on Es. (**B**) Feeding on Ma. (**C**) Feeding on Bp. The color of the nodes represents the name of the phylum, and the label of the nodes represents the name of the genus. The edges between nodes indicate the correlation between genera (red line for positive correlation, green line for negative correlation), and the number of edges connected to nodes reflects the degree of association between genera. Note: Es: *E. grandis × E. urophylla*, Ma: *M. apelta*, and Bp: *B. papyrifera*.

To understand the interactions between the gut microbial communities of *E. signifer* larvae feeding on three different plants, a single-factor correlation network analysis was performed on the top 20 dominant genera of these three groups of samples (Figure 7). The gut microbiota of *E. signifer* larvae feeding on Es (Figure 7A), Ma (Figure 7B), and Bp (Figure 7C) had 19 nodes and 65 edges (46 positive correlations, 19 negative correlations, with a negative correlation ratio of 0.345), 18 nodes and 53 edges (32 positive correlations, 21 negative correlations, with a negative correlation ratio of 0.396), and 19 nodes and 55 edges (26 positive correlations, 29 negative correlations, with a negative correlation ratio of 0.527), respectively. The number of bacterial network nodes in the gut of *E. signifer* larvae feeding on three different plants was almost the same, with the highest number of edges in those feeding on Es being slightly higher; the highest negative correlation ratio was in those feeding on Bp and the lowest was in those feeding on Es.

## 4. Discussion

The insect gut has a diverse array of microorganisms, with bacteria occupying a significant proportion [17]; in detail, the gut bacterial community not only aids herbivorous insects that survive by boring into dry trees in their nutrition metabolism, but also enhances the insect’s defense and detoxification abilities and helps to regulate immune functions [36,37]. For insects, they typically acquire various microorganisms from their environment and food [7], and the type of insect food (e.g., different host preference, wild natural food and artificial diet, etc.) has a significant impact on the community structure of gut microorganisms in insects [38]. In this experiment, we studied the transference and adaption of *E. signifer* to different preferred hosts. The symbiotic bacteria contained in the different hosts will have certain differences, so the gut bacteria will change to different extents due to different host preferences. Unlike the research methods of Wu et al. [18], we transferred the seventh instar *E. signifer* larvae that to adapted Es (the most selected host) to Ma (with a greater feeding preference) and Bp (with no feeding preference).

After the successful transfer, the gut microbial diversity and species composition in Ma and Bp were analyzed together with the *E. signifer* growing in Es. The results showed that there were significant differences in the α diversity and β diversity of the gut bacteria of larvae feeding on the three different plants. This was consistent with the findings of *P. xylostella*, *S. frugiperda*, *H. cunea*, and *Grapholita molesta*, which feed on different hosts and exhibit great differences in the composition of gut bacteria [11,33,39,40]. At the phylum level, the colony composition of herbivorous insects was composed of Proteobacteria, Firmicutes, and Actinobacteria, consistent with *E. signifer*, *Monochamus saltuarius*, *B. mori*, and *Agrilus mali* [41,42,43]. At the genus level, the composition of dominant gut bacteria of Es was not similar to that of Ma or Bp. Interestingly, there was a certain similarity in the composition of dominant bacteria in Ma and Bp, and the dominant bacteria at the genus level in both included *Enterococcus*, *Gordonia*, *Microbacterium*, and *Bacillus*. According to the inter-species difference analysis, the abundance of *Burkholderia*, *Ralstonia*, *Microbacterium*, and *Enterococcus* in the larval gut changed significantly after the host transfer. *Burkholderia* was a common insect-associated bacterium that had the characteristic of being resistant to insecticides and able cooperate with the host to degrade pesticides [3]. *Burkholderia* also had a detoxification function for hosts, which could lead to higher immunity and faster growth and development speed in insects carrying it [44,45,46]. *Ralstonia*, on the other hand, was a destructive soil-borne plant pathogen that has affected the growth and development of more than 50 families and 200 host plants [47]. Although there are few studies recording the discovery of *Ralstonia* in the insect gut, it has appeared in the *B. mori* gut [48]. When changing the host of *E. signifer* larvae, the structure of the dominant bacterial community in the gut was also changed, but because they do not feed on the same host, the similarity of the bacterial communities of the two is not high [49]. However, its specific function in the gut is not known, and it is speculated that it may enter *E. signifer* when they eat the plant. *Microbacterium*, another bacterium found in the larval gut, can produce a broad-spectrum antibacterial activity against primary metabolites and has a strong ability to degrade phenol, which can help the host resist the toxic secondary metabolites of plants [50,51].

*Enterococcus* was abundant in the guts of larvae feeding on Ma and Bp. According to a previous study, *Enterococcus* is common in the guts of herbivorous insects and functions in synthesizing vitamins and amino acids, improving gut immunity, and degrading carbohydrates [52]. Compared to larvae that feed on Es, the bacteria with intergroup differences in the gut of the host feeding on Bp were mostly concentrated in the phylum Actinobacteria. The bacteria of this phylum can provide vitamins for the host and also have certain a antibacterial activity against some plant pathogenic fungi [53]. After the host transfer was completed, the gut bacteria of Ma and Bp, whether at the phylum level or at the genus level, were significantly different from those of Es. However, from the perspective of feeding preference, we did not observe significant differences in the gut bacteria of Ma and Bp. Most studies believe that host switching will cause significant changes in the gut microbiota [39,54,55,56]. The insects were forced to change the environment in their gut in order to adapt to the a environment, so we speculated that the differences in gut microbiota mainly came from host switching [57,58], especially bacteria entering the insects during the consumption of food, settling in the gut [14], which also worked for *E. signifer*.

The microbial interaction network can clearly show the complex interactions between microorganisms [34] and reveal the interaction patterns of gut bacteria in *E. signifer* with different feeding preferences. In this study, we analyzed the interactions between bacterial communities in the guts of *E. signifer* larvae feeding on three different host plants and found most bacteria with communication links. For example, *Burkholderia* was negatively correlated with *Lactobacillus* and positively correlated with *Ralstonia*. Interestingly, *Enterococcus* was a dominant bacterium in the gut of larvae that fed on Es, but it was negatively correlated or unrelated to most bacteria. At the same time, *Enterococcus* in the Ma larvae was positively correlated with *Leucobacter* and negatively correlated with *Gemmobacter*, but in Bp larvae, Enterococcus had no correlation with the two. The composition and metabolic activity of the gut microbiota are influenced by complex interactions between host genetics and environmental factors. As lactic acid bacteria, *Enterococcus* can produce antibacterial substances to change the composition of the gut microbiota [59,60]. *Lactobacillus*, also a lactic acid bacterium, participates in insect sugar metabolism and has the function of synthesizing many carbohydrate-degrading enzymes [61]. It is interesting to note that *Enterococcus* was negatively correlated with most bacteria in the correlation network diagram, which further confirmed this point. Similarly, after the host transfer, the abundance of *Enterococcus* increased significantly, affecting the composition of the gut microbiota; we can further speculate that the differences in gut microbiota mainly come from host transfer. In conclusion, the significant increase in *Enterococcus* abundance is one of the important reasons for the change in *E. signifer* before and after host transfer, which may have a greater impact on gut bacteria. In subsequent research, we should explore the composition of gut microbiota of *E. signifer* eating Ma and Bp natively, observe whether there is a connection with the gut microbiota of *E. signifer* after host transfer, and explore the changes in gut microbiota.

According to the PICRUSt function prediction, the abundance of functional bacterial groups in the primary pathway, except for cellular processes, was consistent with the feeding preferences (Es > Ma >> Bp), mainly enriched in metabolism. Chen supported that the soluble sugar content in wheat leaves was higher than in other plant leaves, and *S. frugiperda,* which feeds on wheat, may need a higher proportion of gut bacteria to assist in carbohydrate synthesis [32]. Wu pointed out that the nutritionally rich artificial feed was balanced and did not require additional synthesis and supply. The amino acid content in plants may not be sufficient to meet the growth and development needs of *P. xylostella* fully; it needs gut bacteria to assist in synthesis [18]. In our previous research, we found that the soluble sugar, reduced sugar, and water content in Bp are all lower than those of Es and Ma (unpublished). After *E. signifer* was transferred to a natural host with lower nutritional components, the larval gut needed a bacterial community to assist the host in completing various life activities such as digestion, absorption metabolism, and stress resistance, thereby changing the diversity of the larval gut microbiota. Therefore, we speculate that the feeding preference of *E. signifer* was also relevant to the difference in nutrients. Furthermore, the abundance of larval gut bacterial communities was mainly concentrated on carbohydrate and amino acid metabolism, and the abundance of bacterial communities in these two metabolic pathways was significantly higher in the gut bacterial communities of *E. signifer* larvae feeding on Bp than those feeding on Es and Ma. This indicated that when *E. signifer* transfer feed on Bp, they need to strengthen the absorption and utilization of carbohydrates and amino acids, to expand the adaptability and ecological range of their food [62,63].

## 5. Conclusions

*E. signifer* is a typical example of a native pest that is able to quickly adapt to exotic trees. This study explored gut bacteria functions for the adaptation of host selection under different stresses (diverse feeding preferences: Es and Ma; no feeding preferences: Bp). The results showed that there were significant differences in the composition of dominant gut bacteria between Es, Ma, and Bp, but without significant differences between Ma and Bp. *Burkholder* and *Microbacillus,* which are detoxification and metabolic pesticides, as well as *Enterococcus* of insect gut probiotics, showed significant changes, and *Enterococcus* can affect the composition of insect gut bacteria. Among them, the significant increase in *Enterococcus* abundance is one of the important reasons for the bacterial changes before and after the host transfer of *E. signifer*. When the connections between the gut bacteria were changed, so did the composition of the gut bacteria. We concluded that the gut bacteria of *E. signifer* were different under different stresses. Furthermore, the feeding preference of *E. signifer* was negatively correlated with the metabolic function of the gut bacteria, showing that the lower feeding preference of *E. signifer*, the higher the metabolic functions in the gut. At the same time, the nutrient content of the host plant affected the feeding preference. In summary, the diversity and species composition of the gut microbiota of *E. signifer* larvae are somewhat related to their preference for different hosts. Further research should explore the correlation between host plant metabolomics and *E. signifer* gut microbiota further, clarify the key functions of gut microbiota, and focus on providing a theoretical basis for the prediction and comprehensive control of potential hosts of *E. signifer*.

## Figures and Tables

**Table 1:** Sequencing information of gut bacteria of larvae feeding on three hosts.

Sample Grouping	Sample	Original Sequence	Number of Valid Sequences	Effective Rate (%)	Average Length (bp)	OTUs Number	Number of Different Classification Orders (Number)				
							Phylum	Class	Order	Family	Genus
Es	E1	48280	47659	98.71	428	170	10	16	38	58	112
	E2	34368	33798	98.34	427	314	14	23	53	87	162
	E3	45944	45460	98.95	429	110	10	18	38	55	79
Ma	M1	33350	31282	93.80	414	350	20	36	87	137	224
	M2	35752	35096	98.17	427	234	19	30	778	127	173
	M3	39971	36412	91.10	422	143	7	13	28	55	91
Bp	B1	33255	32601	98.03	413	174	10	14	4	60	105
	B2	41473	38502	92.84	410	138	12	15	35	62	88
	B3	35377	34264	96.85	417	130	8	10	24	46	80

Note: E1, E2, and E3 were three duplicate samples of Es. M1, M2, and M3 were three duplicate samples of Ma. B1, B2, and B3 were three duplicate samples of Bp. Es: *E. grandis × E. urophylla*, Ma: *M. apelta*, and Bp: *B. papyrifera*.

**Table 2:** Alpha diversity index of gut bacteria of larvae feeding on three hosts.

Sample Grouping	Shannon	Simpson	Ace	Chao1
Es	1.176 ± 0.264 ^B^	0.513 ± 0.121 ^A^	211.651 ± 60.007 ^A^	215.903 ± 60.590 ^A^
Ma	2.376 ± 0.772 ^AB^	0.341 ± 0.191 ^A^	259.654 ± 65.440 ^A^	258.023 ± 65.459 ^A^
Bp	3.105 ± 0.182 ^A^	0.102 ± 0.025 ^A^	159.093 ± 13.660 ^A^	159.718 ± 17.285 ^A^

Note: The data in the table are all “mean ± SE”. Different vertical capital letters indicate that the Alpha diversity index of the gut microbiota of larvae feeding on different plants was significantly different (*p* < 0.05). Es: *E. grandis × E. urophylla*, Ma: *M. apelta*, and Bp: *B. papyrifera*.

## Data Availability

The data presented in this study are available on request from the corresponding author. The data are not publicly available due to their large amount that should not be disclosed.
